# Perfect storm: a scoping review of interventions and preparedness strategies at the intersections of climate change, migrant worker health and health systems

**DOI:** 10.1136/bmjph-2025-003288

**Published:** 2026-06-16

**Authors:** Isabelle Pearson, Karen Lau, Cara Schulte, Nicola S Pocock, Ligia Kiss, Kevin Riley, Chng Saun Fong, Tharani Loganathan, Brad Adams, Andreas Flouris, Sally Hargreaves, Cathy Zimmerman

**Affiliations:** 1 London School of Hygiene & Tropical Medicine, London, UK; 2 St George’s University, London, UK; 3 University of California Berkeley, Berkeley, California, USA; 4 University of California Los Angeles, Los Angeles, California, USA; 5 University of Malaya, Federal Territory of Kuala Lumpur, Malaysia; 6 Department of Social & Preventive Medicine, University of Malaya, Kuala Lumpur, Malaysia; 7 Climate Rights International, Berkeley, California, USA; 8 University of Thessaly School of Health Sciences, Trikala, Greece

**Keywords:** Accidents, Occupational, Environmental Pollution, Environmental Exposure, Public Health, Population Surveillance

## Abstract

**Objectives:**

To map and synthesise evidence on interventions and preparedness strategies addressing climate-related occupational health risks among migrant workers and to identify gaps across five domains: formal health sector; health surveillance systems; regulations or policies; corporate and public procurement and worker, employer, non-governmental organisation (NGO) and academic strategies.

**Design:**

Scoping review conducted in accordance with the Arksey and O’Malley framework and reported using the Preferred Reporting Items for Systematic Reviews and Meta-Analyses (PRISMA) extension for Scoping Reviews.

**Data sources:**

Ovid MEDLINE and Ovid Global Health, searched for peer-reviewed studies published from 1 January 2000 to 28 March 2025, in any language. Expert consultations were used to identify five intervention domains and to supplement database searches.

**Eligibility criteria:**

Peer-reviewed studies reporting on interventions or preparedness strategies at the intersection of climate change, occupational health and migrant worker populations. Studies of any design were eligible. Studies focusing solely on internal migrants or addressing general climate-related health risks without explicit reference to occupational health or migrant workers were excluded.

**Data extraction and synthesis:**

Title, abstract and full-text screening was conducted independently by at least two reviewers, with discrepancies resolved through discussion. Data were charted using a standardised extraction sheet and analysed descriptively and thematically.

**Results:**

19 studies met the inclusion criteria. Most (15) were from the USA; four were from Egypt, Guatemala, UAE and Kuwait. Studies focused primarily on agriculture. Interventions were found for the formal health sector (n=3); regulations or policies (n=4) and workers, employers, NGOs and academics (n=12). We found a critical lack of health sector preparedness to address rising climate-related migration and worker morbidity. Despite the protection potential of policies, regulations and cross-border agreements, only four studies evaluating these approaches were identified. No intervention studies were found for corporate and public procurement or health surveillance. This likely reflects broader gaps in data systems, which rarely collect or disaggregate climate-related health outcomes for migrant workers, especially in low- and middle-income countries.

**Conclusions:**

Health systems remain critically underprepared to detect, foresee and respond to climate-related illness among migrant workers. Migrant-inclusive public health requires surveillance systems that capture this population, stronger protections against exploitative labour conditions that exacerbate climate vulnerability, and demand-side accountability from corporate and procurement actors. Applying just transition principles will promote workers’ participation in decisions shaping their own health, rights and livelihoods.

**WHAT IS ALREADY KNOWN ON THIS TOPIC**

**WHAT THIS STUDY ADDS**

- This scoping review found that of 706 records screened, only 19 studies met inclusion criteria, revealing a critical gap in evidence on preparedness across all sectors. Current evidence is heavily concentrated in heat stress interventions among agricultural workers in high-income countries, with no studies identified for health surveillance or corporate and public procurement domains.

**HOW THIS STUDY MIGHT AFFECT RESEARCH, PRACTICE OR POLICY**

- Findings can guide health systems managers, policymakers and employers to explicitly include migrant workers in climate adaptation plans, occupational health legislation and disaster preparedness frameworks. Results indicate that future research should prioritise low- and middle-income settings, indoor work environments and health surveillance systems and disaggregation of data by migration status, gender, age and occupational sector.

## Background

**Figure 1:**
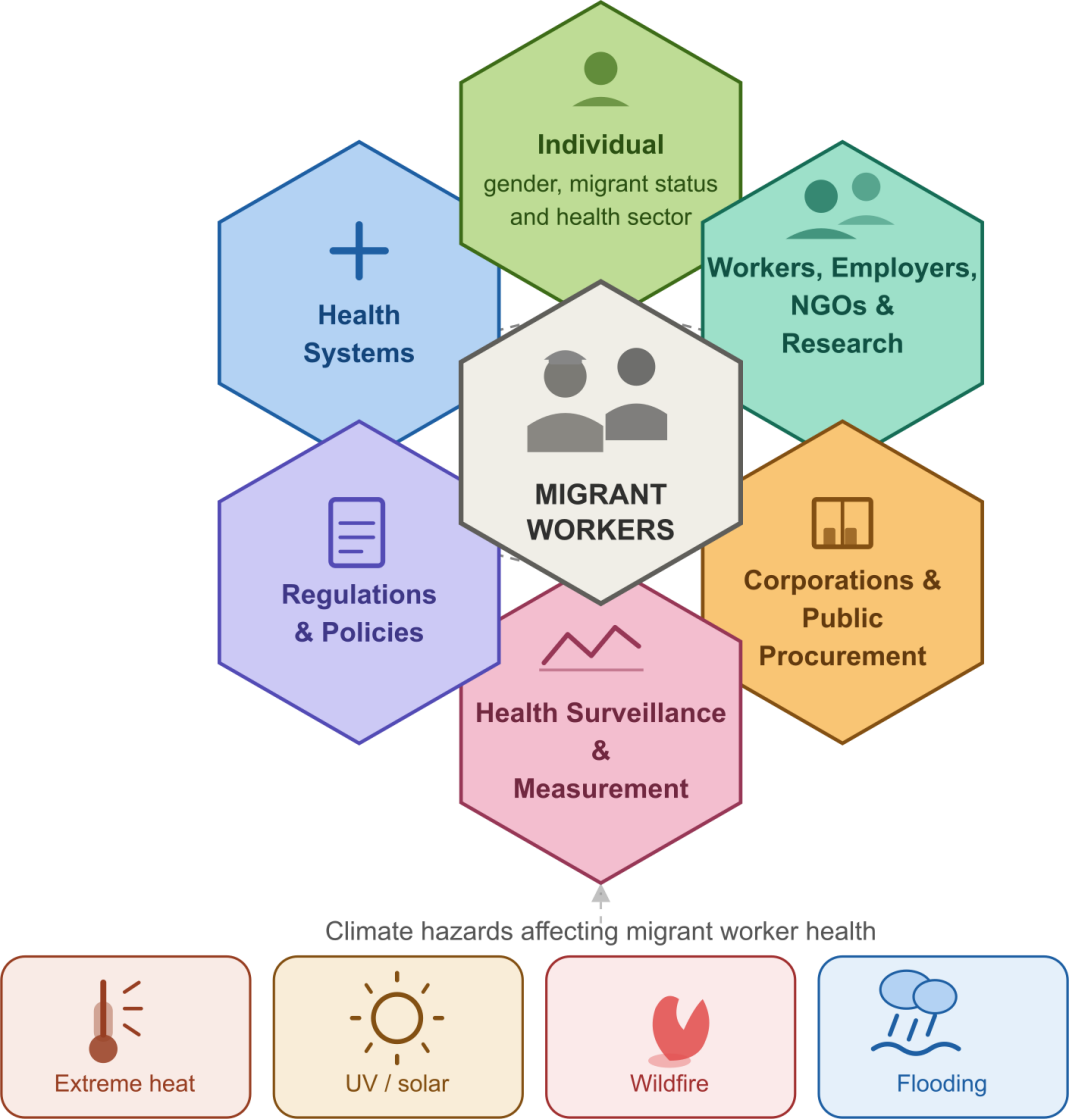
Climate-health preparedness sector domains for migrant worker health. NGO, non-governmental organisation. UV, ultraviolet.

Globally, the migrant labour force plays a vital role in sustaining economies and essential services across sectors such as food supply, transportation, construction, health and caregiving.^1^ Evidence consistently shows that migrant workers operate under disproportionately hazardous conditions and precarious employment terms, ranging from everyday forms of exploitation to severe abuses like human trafficking, modern slavery and 3D jobs (dirty, dangerous and degrading), where they risk acute injuries, disability and chronic illness.^2^,^4^ Climate change is a substantial threat multiplier, compounding existing health vulnerabilities related to labour and migration.^5 6^ Evidence links climate-related effects to a range of health conditions in workers, including cancer, cardiovascular and respiratory diseases, kidney dysfunction and mental health disorders.^7 8^ Yet, to date, there has been little indication how the formal health sector and other sectors will respond to mitigate hazards or treat health outcomes associated with the effects of climate change on work conditions for migrant workers. An overview of the climate-health preparedness sector domains for migrant worker health is provided in figure 1.

Climate-related migration is increasing globally, with individuals relocating both internally and across borders, often concentrating in urban centres or agricultural settings marked by seasonal and circular labour patterns.^9^ Because migrant workers are likely to enter low-wage, precarious jobs—such as gig work, construction, garments, cleaning or call centres—or be pushed into remote areas and high-risk sectors like logging, plantations, mining and fishing, they are at substantial risk of occupational hazards. Beyond the risk of occupational safety and health (OSH) hazards, work arrangements, including both formal and informal, significantly shape health outcomes and access to healthcare.^3^ Studies with trafficked and precariously employed migrants show how exploitative terms and insecure, extractive pay practices (eg, piecework) contribute to poor mental health and social exclusion, especially among minority and temporary workers.^10 11^ A systematic review and meta-analysis demonstrates that migrant workers are consistently exposed to higher risks of occupational injury than native-born workers and receive less occupational and health safety training, including on climate-related hazards.^2^

As the result of extreme climate events and slow onset consequences, both indoor and outdoor workers are exposed to growing climate-related hazards, including heat stress, flooding, landslides, drought, wildfire smoke, ultraviolet (UV) radiation, vectorborne disease (VBD) and disruptions driven by shifting industrial practices.^12 13^ During wildfire events, for example, agricultural and plantation workers are exposed to fine particulate matter, which is associated with acute respiratory effects and, with long-term exposure, elevated mortality.^14^,^16^ These conditions are projected to worsen as climate change advances, exacerbating health inequalities and impacting economic productivity. The Intergovernmental Panel on Climate Change forecasts significant economic losses from heat exhaustion and environmental stressors that directly affect workers, alongside rising strain on healthcare systems.^17^

Reviews link climate change to a wide range of occupational health outcomes, including heat-related illness (HRI), kidney and cardiovascular disease, vectorborne infections, mental illness, higher injury rates and early mortality.^18^ The mental health effects of climate change on migrant workers are particularly significant. For example, chronic exposure to extreme heat has been associated with increased rates of anxiety, depression and psychological distress among outdoor workers.^19 20^ Climate-related disasters such as floods, wildfires and droughts can cause acute traumatic stress and longer-term post-traumatic stress disorder, with migrant workers facing compounded vulnerability due to pre-existing stressors including social isolation, language barriers, precarious legal status and limited access to mental health services.^21 22^ The cumulative effect of occupational climate hazards alongside the psychosocial burdens of migration itself can create a particularly acute mental health burden that current health systems are ill-equipped to address.

Climate risks are also gendered, with women migrant workers facing greater vulnerability to exploitation, sexual violence and harassment and climate-sensitive reproductive and maternal health risks.^23^ For example, for each 1°C increase in annual temperature, physical and sexual domestic violence cases rise by 6.3% in South Asian nations including India, Pakistan and Nepal. (16,17). This relationship is thought to reflect several interconnected mechanisms: heat-related psychological stress, economic instability driven by climate-related livelihood losses and the social disruption of displacement, all of which exacerbate existing power imbalances and increase vulnerability to violence, especially for women and girls.^24^ Furthermore, flooding and other climatic pressures often prompt negative coping strategies, including child marriage, as families seek to mitigate economic and social hardships.^25^

Migrant workers are also among the most exposed to safety and health hazards during extreme weather events. Following severe wildfires, floods and other incidents, migrant workers are frequently hired for tasks such as debris removal, site cleanup and remediation or reconstruction, often working outside of more formal disaster response and recovery networks and without the level of training and protections that those networks might afford.^26^

Despite their disproportionate presence in high-risk, poorly regulated sectors, migrant workers remain largely unprotected by national systems, particularly health systems that are underprepared for the growing strain of climate-related illness and injury. Few health strategies acknowledge that these fundamental social and environmental determinants of health—represented as worsening work conditions and outcomes among migrant workers—will impact national infrastructure and local health services, as well as global health.^27^ Yet, this protection gap is not inevitable: experts have emphasised that climate-related health risks are foreseeable and can be mitigated through proactive preparedness and adaptation using tools such as strategic foresight.^28^ For instance, techniques like strategic foresight, which offer structured, forward-looking practices to consider multiple plausible futures, can help health systems, policymakers and employers anticipate and prepare for the compounding effects of climate-driven hazards on migrant worker health, thereby promoting more proactive and equitable policy and planning responses.

Furthermore, while many climate-health interventions target agricultural work settings, there is less evidence of other intervention models or studies that incorporate social determinants, such as living conditions, legal exclusions, language and literacy—a gap this review seeks to address.^3 29^ Current reviews tend to examine either climate change and occupational health, or migrant worker health in isolation, rarely the intersection of all three: climate change, migrant labour and health system preparedness. Without a clearer picture of what interventions and preparedness strategies currently exist, health systems, policymakers and employers will have difficulty identifying where action is most urgently needed or where evidence is weakest. Migrant workers sit at precisely this intersection: disproportionately exposed, structurally excluded from protections afforded to citizen workers, and largely absent from climate adaptation frameworks. This scoping review therefore maps current evidence on interventions and preparedness strategies at the intersection of health, migrant labour and climate change, with the aim of identifying gaps, informing and making the case for migrant-inclusive climate health preparedness.

## Methods

This exploratory scoping review maps the available peer-reviewed literature on interventions and preparedness strategies addressing climate-related occupational health risks among migrant worker populations. The review was framed using the Population, Concept and Context (PCC) framework recommended for scoping reviews: Population—international migrant workers; Concept—interventions and preparedness strategies addressing climate-related occupational health risks; Context—occupational and health system settings globally. The methodology used in this scoping review followed the Arksey and O’Malley approach^30^ and the study was reported according to the Preferred Reporting Items for Systematic Reviews and Meta-Analyses (PRISMA) guidelines for Scoping Reviews (see online supplemental materials for PRISMA-ScR Checklist).^31^ A search strategy was developed using keywords related to five key concepts: migrants, workers, climate change, health and preparedness (online supplemental material). Searches were conducted in two bibliographic databases (Ovid MEDLINE and Ovid Global Health) for studies published from 1 January 2000 to 28 March 2025, in any language. Additional relevant studies (n=4) were identified and included using an iterative approach, including consultations with subject matter experts who were subsequently invited to contribute as co-authors of this manuscript and are listed in the author list. This approach is consistent with the Arksey and O’Malley scoping review methodology. Based on these expert consultations, five domains were identified to categorise the interventions: formal health sector; health surveillance systems; regulations or policies; corporate and public procurement; and workers, employers, non-governmental organisations (NGOs) and academics.

Title, abstract and full-text screening were conducted independently by at least two reviewers (CZ, KL) with discrepancies resolved through discussion. Rayyan (https://rayyan.ai) was used to facilitate deduplication of records and screening of articles. Studies were eligible for inclusion if they: (1) focused on international migrant workers; (2) reported on an intervention or preparedness strategy addressing climate-related occupational health risks and (3) were of any design, including qualitative, quantitative, mixed methods and reviews. Studies were excluded if they focused solely on internal migrants or addressed general climate-related health risks and preparedness without explicit reference to occupational health or migrant worker populations. Data from studies that met the eligibility criteria were extracted into a data extraction sheet in Microsoft Excel which captured key study characteristics and findings, and were analysed using descriptive and thematic analysis. Data charting was cross-checked by two reviewers (CZ and KL).

Where relevant, we also draw on findings from studies on occupational health or climate-related worker protections that did not meet the full inclusion criteria for the scoping review, where these offered illustrative examples or important contextual evidence.

### Patient and public involvement

No patients or members of the public were involved in the design, conduct, reporting or dissemination of this scoping review.

## Findings

**Figure 2:**
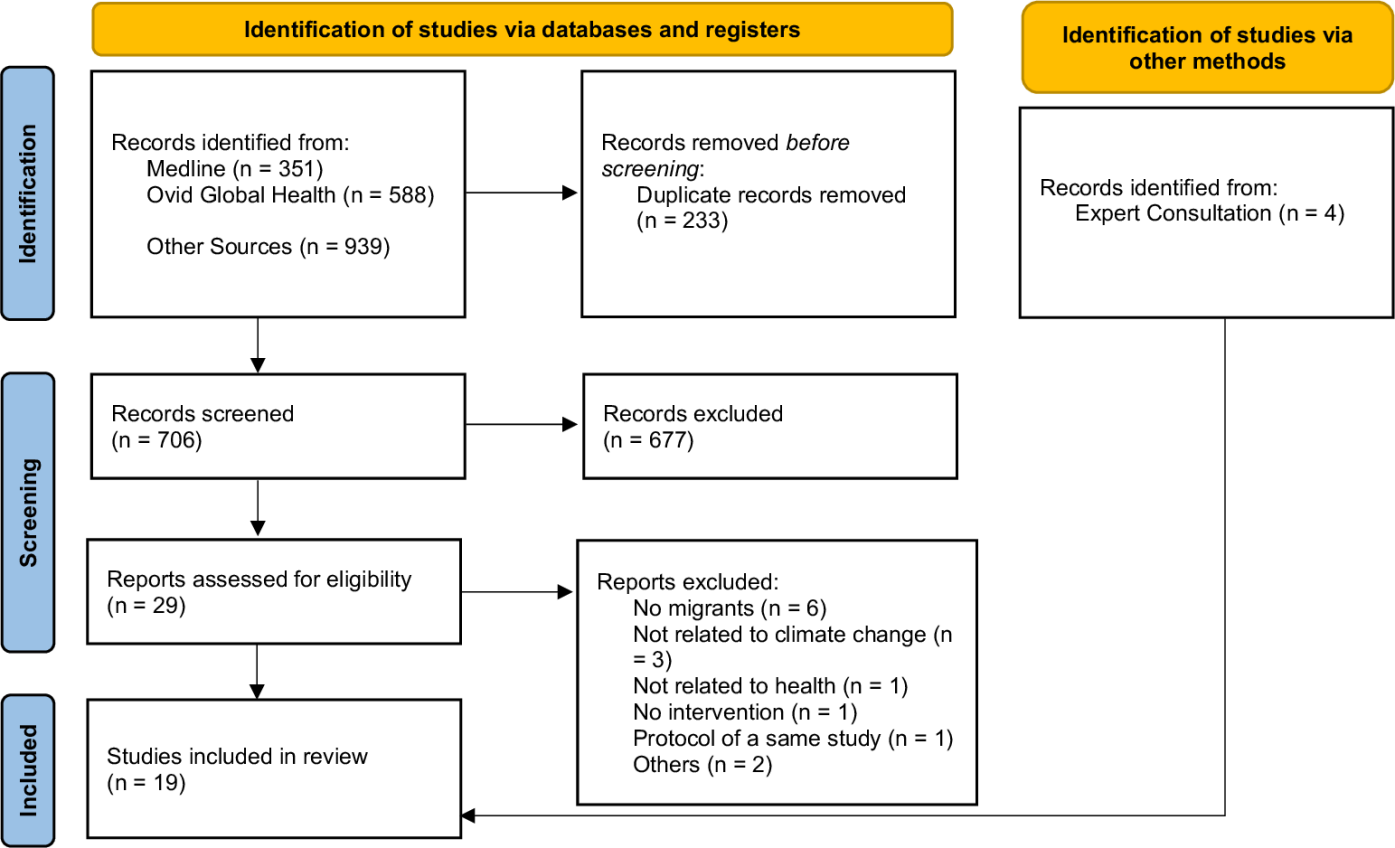
PRISMA flow chart. PRISMA, Preferred Reporting Items for Systematic Reviews and Meta-Analyses.

Of 706 non-duplicate records, we included a total of 19 (quantitative, qualitative and mixed-methods) studies. Fifteen of 19 studies were conducted in the USA, and four were conducted in Egypt, Guatemala, UAE and Kuwait (figure 2). 17 studies focused on the agriculture sector. Based on our search criteria to explore various stakeholder-driven interventions, identified studies described interventions initiated or delivered by: formal health sector (n=3); regulations or policies (n=4); and workers, employers, NGOs and academics (n=12). No intervention studies were found for: health surveillance systems or corporate and public procurement.

The selected preparedness categories below are informed by Benach *et al*’s macro and micro frameworks on work-related influences on health.^32^ The macro level highlights how power dynamics shape labour markets and welfare through regulation, bargaining and social policy, while the micro level addresses how employment and working conditions affect individual health. Peer-reviewed studies were identified for three of the five climate-health preparedness domains searched (see tables 1–3):

1. Health system and service responses to occupational and climate risks and outcomes among migrant workers (n=3) (table 1).
2. Regulations and policies for climate-responsive OSH (n=4) (table 2).
3. Worker, employer, organised labour and non-governmental strategies for climate-safe work conditions (n=12) (table 3).

**Table 1:** Health system and service responses to occupational and climate risks among migrant workers (n=3)

Authors and year	Country	Population	Climate exposure	Intervention	Study design	Sample size	Key findings
Brady *et al*., 2024^35^	USA	Nursing students (BSN, MSc, DNP)	Heat	Educational intervention on heat-related illness first aid for agricultural workers	Pre–post test	n=55	BSN student scores improved from 61% to 93%; graduate students from 69% to 96%
Joubert *et al.*, 2011^36^	UAE	Construction workers, supervisors, HSE personnel and employers/owners	Heat	Media campaign: Safety in Heat programme	Process evaluation (input, process, outcome)	465 companies (815,000 workers)	Two companies reported 79.5% and 50% declines in heat-related cases
Shaban *et al*., 2024^37^	Egypt	Elderly agricultural workers (≥60 years; 79 male, 41 female)	Heat	6-week geriatric nursing-led programme: hydration, rest breaks, reflective clothing, shade and heat strain response training	Community-based quasi-experimental design	n=120 (intervention+control)	‘Safe’ heat strain category increased from 26.7% to 45%; ‘Danger’ category decreased from 25% to 15% in the intervention group

BSN, Bachelor of Science in Nursing; DNP, Doctor of Nursing Practice; HRI, heat-related illness; HSE, Health, Safety and Environment; MSc, Master of Science in Nursing.

**Table 2:** Regulations and policies for climate-responsive occupational safety and health (n=4)

Authors and year	Country	Population	Climate exposure	Intervention/policy	Study design	Sample size	Key findings
Alahmad *et al.*, 2023^44^	Kuwait	Non-Kuwaiti migrant workers in construction and hospitality	Heat	June–August ban on midday outdoor work	Secondary analysis of 5 years (2015–2019) of occupational injury reports	96% of injuries were from non-Kuwaiti migrant workers	Higher temperatures (≥39.4°C) associated with increased injury risk (RR up to 1.48) during the summer months despite the ban
Iyore *et al*., 2025^43^	USA	Migrant agricultural workers in North Carolina	Climate-related disasters	Emergency preparedness best practices: Spanish materials, vulnerability mapping, worker input	Quantitative content analysis of 47 emergency preparedness plans from 41 counties in eastern North Carolina	47 plans evaluated	Most plans excluded agricultural workers and lacked best practices addressing climate-related vulnerabilities
Jackson *et al*., 2010^42^	USA	Agricultural workers in California and Washington	Heat-related illness	Review of implementable heat stress regulations	Review (prepared for Agricultural Safety and Health Council of America/National Institute for Occupational Safety and Health Conference)	N/A	Standards pose compliance challenges and may offer uneven protection across agricultural settings
Langer *et al*., 2021^48^	USA	Latino farmworkers (36% women; mean age 38.8)	Heat (core body temperature, wet-bulb globe temperature)	California Division of Occupational Safety and Health heat regulations: compliance and effects	Cross-sectional study	n=507 farmworkers on 30 farms	Heat-related illness training and hydration were inadequate; elevated core temperature linked to male sex, higher wet-bulb globe temperature, body mass index and work intensity

N/A, not applicable; RR, relative risk.

**Table 3:** Worker, employer, NGO and academic strategies for climate-safe work conditions (n=12)

Authors and year	Country	Population	Climate exposure	Intervention/strategy	Study design	Sample size	Key findings
Bethel *et al.*, 2017^101^	USA	Latino farmworkers (56% male; mean age 36)	Heat	Worker-adopted cooling: breaks, hydration, headwear and clothing	Cross-sectional study	n=197 (in Oregon and Washington)	Cooling practices varied by state; gaps in employer-provided shade and hydration identified
Brown *et al.*, 2006^62^	USA	Farmworkers in California	Heat and other non-climate occupational exposures	Union organising and collective bargaining for California Division of Occupational Safety and Health heat stress standard	Historical review	N/A	Unions prioritising health and safety in organising efforts; collective action linked to policy change
Chavez Santos *et al.*, 2022^63^	USA	Outdoor adult farmworkers in Washington (male; aged 18+)	Heat (exertion levels, wet bulb globe temperature)	Participatory heat education and supervisor heat decision-support app (HEAT intervention)	Parallel comparison group intervention study	n=75 (37 intervention, 38 comparison)	Higher heat-related illness symptom risk linked to older age, higher heat index, longer agricultural tenure, limited toilet access and non-H-2A* status
Chicas et al., 2021a^102^	USA	Hispanic outdoor farmworkers in Florida (66% female; mean age 42)	Heat	Workplace personal cooling gear to prevent heat-related illness without disrupting work routines	Qualitative interview study	n=61	Workers reported acceptability of cooling gear; practical barriers to consistent use identified
Chicas et al., 2021b^103^	USA	Mixed outdoor agricultural workers in Florida (66% female; aged 18–54)	Heat (core body temperature threshold ≥38.0°C)	RCT with four groups: no intervention, cooling bandana, cooling vest or both	RCT	n=84	Cooling bandana reduced odds of exceeding core temperature threshold; vest and combined use showed no clear benefit
Dillane *et al.*, 2020^64^	USA	Worksite-level analysis in North Carolina (no individual workers)	Heat stress	Assessment of Occupational Health and Safety Administration–National Institute for Occupational Safety and Health Heat Safety Tool app reliability	Field monitoring study	2 agricultural sites	App reliably identified minimal risk (60%–100%) but less accurate under high heat stress and variable workloads
Luque *et al.*, 2019^65^	USA	Hispanic farmworkers (aged 19-66; Florida and Georgia border; mostly Mexican-born; 60% male) and crew leaders (aged 26-69, 50% male)	Heat	Training crew leaders to use Occupational Health and Safety Administration heat safety tool app	Cross-sectional study	6 crew leaders; 101 farmworkers	70% of farmworkers reported drinking water every 30 min; 77% used natural shade; and adaptive cooling strategies were used such as wet hats (11%), hydration salts (32%) and altering work/rest routines (21%–66%).
Marquez *et al.*, 2023^104^	USA	Latinx farmworkers (65% male; aged 18–64)	Heat	Spanish/English participatory, culturally tailored heat education	RCT	n=83 (40 intervention, 43 control)	Knowledge scores significantly improved in the intervention group compared with control with a pre-post increase of 1.6 vs 0.41 (p=0.04).
Mizelle *et al*., 2024^51^	USA	Latino male migrant farmworkers in eastern North Carolina (mean age 38)	Heat	Backpack hydration systems	Cross-sectional study	n=47	74% drank more water; 79% drank more frequently with backpack system
Riley *et al*., 2012^52^	USA	Community and worker leaders from industries employing large migrant worker populations (Los Angeles)	Heat	Peer-led outreach using community health promoters (promotores) to educate workers on California heat illness regulations	Qualitative intervention evaluation	n=159 community leaders	Peer trainers filed California Division of Occupational Safety complaints leading to citations in carwash and warehouse industries
Sorensen *et al*., 2020^53^	Guatemala	Sugarcane fieldworkers (mean age 28.6)	Heat	Increased hydration (electrolytes and water) plus structured rest and shade	Cohort study	n=483	Kidney function improved or stabilised: 85% with acute decline showed improvement; 74% showed reduced kidney injury markers
Stoecklin-Marois *et al.*, 2013^105^	USA	Latino farmworkers in California (64% Mexican-born; 263 men, 211 women; mean age 40)	Heat	Heat illness training, employer-provided and worker-provided beverages, shaded break areas, and symptom-triggered response	Follow-up interview of random community-based sample	n=474	Documented multiple health outcomes (respiratory, injury, mental health, reproductive); intervention coverage gaps noted

* H-2A is a United States federal programme where agricultural workers can be hired on temporary work permits from other countries.

HEAT, Heat Education and Awareness Tools; N/A, not available; NGO, non-governmental organisation; RCT, randomised controlled trial.

**Figure 3:**
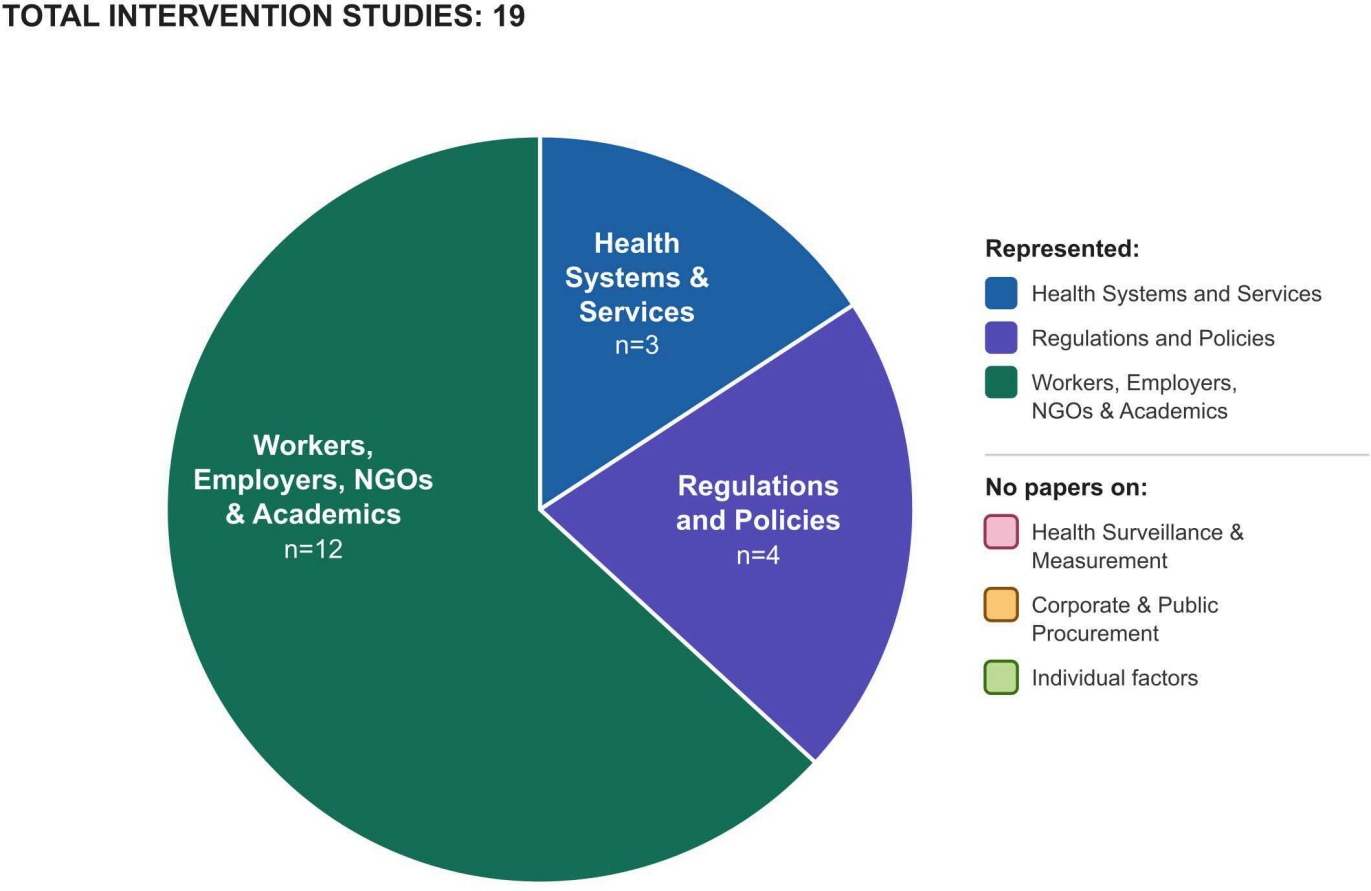
Overview of number of papers identified for each domain. NGO, non-governmental organisation.

We also sought interventions in two other domains: (1) health surveillance systems and (2) corporate or public procurement sectors, but no studies were identified—despite their potential to safeguard migrant health amid intensifying climate events, rising labour migration and escalating occupational risks (see figure 3).^33^

### Health System and Service Responses to Occupational and Climate Risks and Outcomes among Migrant workers

The health sector and the health workforce have an important role to play in strategies to protect migrant workers in the context of climate change, including preventing, detecting and managing risks such as heat illness, dehydration and respiratory conditions.^34^

This review identified three peer-reviewed studies on the health sector’s role in climate response designed to reach migrant workers. The first study evaluated heat illness first aid training for 55 nursing students serving farmworker communities, reporting a significant increase in knowledge.^35^ A second found that, following implementation of the Health Authority’s Safety in Heat programme, two large companies in Abu Dhabi reported substantial declines in heat-related cases—one with a 79.5% decrease per 1000 workers, and the other with a 50% reduction per 100 000 work hours.^36^ A third conducted a quasi-experimental trial of a geriatric nurse-led programme among 120 elderly agricultural workers (>60 years), finding improved safety practices and reduced heat risk in the intervention group.^37^ However, no studies were found on broader health system strategies to reach or treat migrant workers, such as emergency department preparedness, coordination with employers or unions, targeted occupational risk screening and delivery of heat-stress protocols, inclusion in medical school curricula or migrant-specific public health alerts.^38^

Despite finding examples of mobile health clinics that reach migrant workers, there were no studies on formal health sector interventions with climate-related health outreach for migrant workers.^39^ Similarly, while literature shows that some emergency departments have introduced specialised triage pathways for heat-related illness (HRI),^40 41^ our review found no studies describing adaptations for diverse migrant worker populations—such as multilingual heat alert protocols or post-disaster care.

### Regulations and policies for climate-responsive occupational safety and health

Climate-related, migration-related, labour-related and health-related mandates are rarely covered under the same policy, law or regulation. Findings from this review confirm the scarcity of analyses of policies, regulations or laws specifically addressing climate-related health risks for migrant workers, despite the potential of national, regional and bilateral frameworks to promote worker entitlements and health protections. Four studies on policies or regulations were identified: three in the USA and one in Kuwait. One US study examined the challenges of applying generic heat regulations to agricultural settings.^42^ A second US-based study conducted a content analysis of emergency preparedness plans, reviewed best practices, such as Spanish-language materials and outreach to migrant agricultural workers.^43^ The third US study examined compliance with and effectiveness of California Occupational Safety and Health Administration (OSHA) regulations to reduce HRI. The Kuwait study investigated the influence of the summertime ban on midday work on workers’ injury rates.^44^

However, studies commonly indicate effectiveness is often limited by weak enforcement, scarce inspections, employer non-compliance and workers’ fear of retaliation—especially in informal or unregulated sectors.^445^,^47^ Even where climate-responsive occupational safety and health (OSH) policies exist, enforcement remains uneven. California’s heat illness prevention standard (Title 8, Section 3395) mandates rest, shade and water during extreme heat, regardless of immigration status. Yet, as Langer *et al* found, workers still faced elevated HRI risk due to high work rates and temperatures—even when regulations were followed. Moreover, non-compliance is widespread, and national standards often lag behind current climate science.^48^

This review found no studies examining cross-border migration policies in destination countries that incorporate climate-related worker protections, even where equitable labour standards exist.^49^ Similarly, we found no evidence that sending country mechanisms like the Philippines’ Overseas Workers Welfare Administration—which offers insurance, medical aid and repatriation—include climate-related protections, despite many migrant workers being employed in high-risk sectors such as fishing and construction.^50^ Likewise, papers on regional frameworks, such as the EU’s cross-border healthcare directive, the European Health Insurance Card, the Mercosur Residence Agreement and Association of Southeast Asian Nation’s (ASEAN) migrant rights declaration, did not recognise climate-related exposures, despite their aim to secure access to health services and standardise entitlements across borders.^51^,^53^ Bilateral labour agreements have also shown limited success in addressing climate-related risks. Evidence from Pakistan and Qatar, despite Qatar’s 2020 heat regulations, highlights how poor housing, weak enforcement and restrictive legal systems undermine impact.^54^ The International Labour Organization (ILO) identified 21 countries with emergency protocols on heat at work.^55^ Most recently, Japan adopted heat protocols to protect workers, including provision of emergency transport for heat-affected workers, and severe financial penalties for non-compliant employers.^56^

Similarly, our review indicated that national climate disaster plans frequently overlook migrant workers. Emergency protocols, evacuation plans and postdisaster guidelines often fail to include migrants. Notable exceptions include the UK’s high financial penalties for OSH violations and Malaysia’s OSH Act 1994, which enforces criminal liability for employer negligence.^57 58^ In California, emergency wildfire regulations require employers to provide masks when the Air Quality Index exceeds safe thresholds.^59^ In the USA, only six states have heat stress regulations and few protect indoor workers.^60^ Though provisions like ‘weather and safety leave’ exist (eg, US Title 5, Section 6329c),^61^ few frameworks address long-term risks or compensation.

### Worker, employer and non-governmental strategies and academic research for climate-safe work conditions

The first line of occupational protections for workers’ health and safety are workplace measures. This review identified 12 papers that described interventions or strategies initiated by either workers (n=2), both workers and employers (n=1), NGO or community organisations (n=2), or were conducted as a research project (n=7).

The literature includes studies on workplace protocols, worker-adopted personal protective equipment (PPE), NGO-led interventions and academic initiatives. Evidence suggests that, in the absence of employer protections or subsidised PPE, migrant workers often initiate their own informal climate-related health measures. Two studies found that provision of a backpack hydration system or education on increasing hydration (electrolytes and water) in addition to structured rest and shade increased agricultural workers’ water intake and stabilised or improved the trajectory of kidney function decline among all workers irrespective of baseline kidney function.^51 53^ Two studies based in the USA found that migrant worker participatory approaches were effective in improving health and heat safety standards at the workplace, including a community-based ‘train the trainer’ programme by University of California, Los Angeles (UCLA) Labor Occupational Safety and Health Program and labour organising efforts through unions.^52 62^ Three studies assessed the effectiveness of using technology assistance, including the HEAT mobile application and the OSHA-NIOSH Heat Safety Tool mobile app, indicating some evidence of usefulness in risk identification among agricultural workers.^63^,^65^

While most climate-related protections target outdoor workers, emerging research highlights similar risks for indoor workers facing heat stress and poor air quality. A 2025 scoping review found that effective mitigation requires ventilation technologies, adjusted schedules, breathable PPE, training and strong workplace policies.^66^ Additional evidence from modelling and field studies supports rooftop cooling. For example, a Seoul study found that cool roofs reduced surface temperatures by 5.6°C and indoor temperatures by 0.56°C, with effectiveness shaped by outdoor temperature, sunlight and humidity.^67^

In wildfire-prone areas, tailored medical protocols help prevent the worsening of asthma and cardiovascular disease.^68^ Research also suggests that employers are well positioned to provide early onsite or community-based health screening and prompt referral for treatment of climate-sensitive illnesses.^69^ A literature review of occupational case reports additionally describes the climate-driven spread of vector-borne diseases (eg, malaria, dengue, zika) and zoonotic diseases (eg, hepatitis E, West Nile Virus), especially in certain occupational settings.^70^ To protect workers, employers can, for instance, limit outdoor work during peak mosquito activity times (eg, dawn and dusk), eliminate standing water around worksites and provide screened rest areas among especially at-risk workers (eg, agricultural workers, animal handlers, soldiers).

Employers can play a key role in emergency preparedness by establishing protocols and training workers to respond to climate-related disasters, including evacuation and communication plans, as recommended by US OSHA.^71^ During extreme events or recovery work, they may also provide hazard pay, bonuses or relocation support.

### Other sector interventions

Despite their potential role, we found no studies led by health surveillance bodies or corporate and public procurement actors. Because of the importance of their potential actions, we sought key examples of intervention approaches that could be adapted to operate at the nexus of health preparedness, migrant labour, occupational health and safety and climate change, presented below.

### Surveillance and measurement of Climate-Related Health Risks and Outcomes among Migrant Workers

To date, where climate effects are monitored, data are rarely collected or disaggregated to measure the effects on occupational health among migrant workers, particularly those in the most at-risk sectors, or informal and undocumented workers—especially in low- and middle-income countries.

Public health data seldom include occupational information, limiting the ability to link work and health outcomes—especially for migrant workers, who are often missed by surveillance systems due to limited healthcare access and underutilisation.^72^ Yet, studies have suggested that health workers are not confident in their ability to assess the potential impacts of climate change.^73^ While climate-health indicators^74^ and toolkits to promote climate readiness are emerging,^75^ few specifically describe mechanisms or appropriate indicators for surveillance of the diverse migrant labour workforce. Climate induced vector-, food- and water-borne diseases and temperature-related health outcomes have been suggested as high priority health indicators in a recent systematic review.^76^ Using heat stress as an example, existing International Classification of Diseases, 11th Revision (ICD-11) codes can be used to document health-related outcomes of heat stress at work (see table 4). The Wet Bulb Globe Temperature (WBGT) index is the most widely used global indicator for assessing environmental heat stress, incorporating temperature, humidity, wind speed and thermal radiation, along with occupational exposure limits that account for work intensity and PPE.^55^ WBGT measurements can be aggregated for regional and national surveillance of heat stress, such as in heat stroke early warning systems.^77^ Linking patient records coded for HRI with WBGT data from migrant labour-intensive industries in specific regions could serve as a prototype for heat stress surveillance among migrant workers. Developing such systems will require the inclusion of appropriate indicators to capture migrant status in occupational health data.^78^

**Table 4:** Globally recognised measures for monitoring heat stress and outcomes

Example measure	Unit of data capture	Surveillance implication	Example standard/code	Source
Environmental heat stress				
Wet Bulb Globe Temperature Index (WBGT) and recommended Occupational Exposure Limits for heat exposure depending on work tasks, intensity and acclimatisation status.^106^	Workplace	Workplace data can be aggregated at regional/sector levels (eg, similar to Heat Stroke Alert warning systems, based on WBGT data from different cities/locations, eg, Japan)^77^ in industries that are heavily reliant on migrant workers.	NIOSH recommended standard for acclimatised workers: Light work: WBGT up to 86°F (30°C) Moderate work**:** WBGT up to 82°F (28°C) Heavy work: WBGT up to 77°F (25°C)	US OSHA, NIOSH, ACGIH^107 108^
Health outcomes				
ICD-11 codes^109^	Patient	Analysis of patient records, combined with migrant status, occupation/economic activity ICD extension codes.	NF01 Effects of heat NF01.0 Heat stroke NF01.1 Heat syncope NF01.2 Heat exhaustion due to fluid depletion NF01.3 Heat fatigue, transient	ICD-11^109^

ACGIH, American Conference of Governmental Industrial Hygienists; ICD-11, International Classification of Diseases, 11th Revision; NOISH, National Institute for Occupational Safety and Health; US OSHA, United States Occupational Safety and Health Administration; WBGT, Wet Bulb Globe Temperature Index.

### Corporate and public procurement actions for climate-safe workplaces

Multinational corporations (MNCs), state-owned enterprises (SOEs) and public procurement systems have substantial potential to reduce climate-related risks for migrant workers by setting and enforcing climate-responsive labour standards. However, this review found no peer-reviewed studies on corporate-led, SOE-led or procurement-led strategies specifically addressing climate-related health risks among migrant workers. Despite a growing body of literature on sustainable business practices and established frameworks—such as the UN Guiding Principles on Business and Human Rights and Organisation for Economic Co-operation and Development (OECD) Guidelines—there remains a notable evidence gap on concrete strategies to mitigate occupational climate risks for any workers, migrant or otherwise.^79 80^

In the grey literature, a widely cited example of combined corporate-government-NGO strategy comes from FIFA World Cup construction in Qatar, where MNCs—working with the ILO and Supreme Committee—implemented measures like shaded rest areas, hydration stations, medical screenings and ‘StayQool’ suits.^81^ These measures informed legislation mandating work stoppages when the WBGT exceeds 32.1°C, intended to improve work conditions at large sporting events.^55^ However, Qatar drew widespread criticism as these advancements were introduced long after heat-related OSH violations were reported, including migrant worker deaths and hospitalisations during World Cup construction projects.^82^

More commonly, MNCs rely on supplier codes of conduct, which typically lack enforceable climate protections and have limited accountability across global supply chains.^83^ In the fashion sector, where rising temperatures pose serious health risks to indoor workers, few brands have included heat stress mitigation protocols in supplier standards.^84 85^ Even where such standards exist, implementation is inconsistent, especially in regions with weak labour enforcement.^86^ Similarly, while public procurement directives increasingly include human rights and climate considerations, they often omit explicit safeguards for worker health in climate-sensitive contexts.^87^

Voluntary third-party audits and social responsibility agreements are often promoted as solutions, yet their effectiveness remains widely questioned and few include explicit climate-related provisions.^88^ However, there is growing support for worker-driven social responsibility, which places workers at the centre of designing, monitoring and enforcing labour protections—including those related to climate risks.^89^ Yet, little is known about how worker-driven social responsibility mechanisms or worker-led monitoring influence climate-related exposures beyond the agricultural sector.

During climate disasters, although emergency protocols like early warning systems and disaster relief funds are widely recognised as valuable, there is little evidence that MNCs, SOEs, or government agencies adopt them or require suppliers to adopt them. In some cases, policies may heighten risk: for example, California’s Agricultural Pass (Ag Pass) system allows farmworkers to re-enter wildfire evacuation zones to continue ‘essential’ labour, with some employers offering hazard pay that may incentivise workers—often migrants—to remain in dangerous conditions.^15^

## Discussion

This review aimed to identify what we know about system readiness to protect the health of migrant workers amidst escalating climate-related risks. The most worrisome finding of this review is the seeming lack of health sector preparedness for the anticipated rise in climate-related labour migration and associated morbidity among migrant workers. The scarcity of published interventions and health surveillance systems led by the formal health sector highlights a critical gap in preparedness. The formal health sector and health surveillance systems are promising starting points to design and deliver health interventions, as suggested by a previous review of health promotion programmes addressing climate change risks.^90^ Among the findings, this review points to the potential role of medical staff in addressing the additional climate-related health risks among migrant workers. The role of health professionals suggests the value of integrating climate-related OSH into medical and nursing education, especially emergency medicine, where the most vulnerable workers are most likely to seek care. This finding aligns with broader evidence that health systems globally remain underprepared for climate-related health impacts, with Romanello *et al* identifying critical gaps in health system adaptation across all income settings.^91^ The absence of health surveillance systems that disaggregate data by both occupation and migration status represents a foundational gap. Without these data, the burden of climate-related illness among migrant workers will remain largely invisible to planners and policymakers.

We did not identify specific health surveillance and measurement approaches that cover climate, migrant labour and health, despite the potential of these data to improve risk identification and reduction. However, strong analysis of these data will require methods to estimate the proportion of outcomes attributable to climate change and to project future impacts, which often relies on local capacity for advanced analysis or modelling. More robust and comparable datasets, including disaggregated data on migrant worker morbidity and mortality, will also be essential to assess the global burden of low-wage, exploitative working conditions and their associated climate-related health impacts.

Simultaneously, our review found that current climate-health literature is narrowly focused on heat stress among outdoor agricultural work, primarily in high-income countries. Far less attention is given to other outdoor sectors, indoor workers, and a broader range of climate-related exposures, such as air pollution, wildfire smoke, vector-borne and water-borne diseases, and post-disaster hazards. Moreover, little intervention evidence comes from low- and middle-income countries, where heat is more often framed as a migration driver than as an occupational health concern at destinations.^92 93^ This geographic skew is consistent with patterns identified in broader occupational health research, where evidence from low- and middle-income countries is systematically underrepresented despite these settings bearing a disproportionate share of climate-related labour burdens.^94^ Researchers, funders and journals should prioritise studies from these settings, particularly those examining indoor workers, postdisaster recovery workers, and those exposed to vector-borne diseases and wildfire smoke. Additionally, our review showed that despite growing corporate and state sustainability messaging, there appears to be little evidence of enforceable standards or accountability mechanisms to mitigate occupational climate risks across supply chains.

While not a focus of this review, evidence on the health impact of anti-immigrant policies and legislation continually shows how they impede access to healthcare and social protections, particularly for undocumented or seasonal workers.^95^ Similarly, migrant workers are commonly operating under exploitative and precarious employment structures, which can be determinative of risk exposures. The growing gig sectors and contract work, which provide few health protections for workers, may pose increasing threats to wider public health, for both migrant and non-migrant workers. For example, research in Vietnam with app-based couriers, highly climate-exposed urban workers, many of them migrants, found that precarious gig economy employment structures increased both health risks and climate vulnerability.^96^ Similarly, low-paying piece-rate systems incentivise excessive hours and physical exertion, further reducing workers’ resilience to climate-related hazards.^97^ These structural vulnerabilities call for policy responses that go beyond workplace-level interventions. For example, with the growth of the gig economy, policymakers should consider setting occupational health and safety protections explicitly for the gig economy and piece-rate workers, regardless of employment classification. Specifically, these regulations should ensure that climate adaptation frameworks address labour market precarity as a determinant of climate vulnerability.^98^

Although migrant workers are often on the frontlines of climate-related harm, they are rarely included in climate preparedness and adaptation planning. Dialogues on *Just Transition for Workers* have advanced, but rarely address occupational health and safety equity, focusing instead on labour market impacts and the economic challenges of transitioning to a low-carbon economy.^99^ Yet, the moral and practical case for including migrant workers is compelling and urgent. The COVID-19 pandemic revealed how inadequate public health preparedness for migrant workers can rapidly disrupt essential services, transform entire industries and produce cascading effects on workers’ health and the wider economy.^1^ Economic models have shown that the effects of climate change on workers are among the most important drivers of the total economic costs of climate change.^100^ Scholars have argued that promoting safer conditions for climate-related migration is a public health imperative to mitigate harm and prevent loss of life. For policymakers, this means that migrant-inclusive climate adaptation is not merely a matter of equity, it is an economic and public health necessity. We therefore call on governments, international bodies and employers to explicitly include migrant workers in national climate adaptation plans, Just Transition frameworks and occupational health and safety legislation, ensuring that workers themselves have a meaningful voice in the decisions that shape their health, rights and livelihoods.

This review calls on the global community—and the health sector in particular—to be better prepared to protect migrant workers from climate-driven health risks. Tools such as strategic foresight offer a valuable approach to anticipate and shape responses by systematically exploring multiple plausible futures of work and migration and climate-related occupational risk.^28^ In the OSH context, forecasting risks and outcomes can help counter common decision-making pitfalls, such as overestimating short-term change and underestimating long-term disruption. By broadening what health systems consider relevant and grounding strategies in the experiences of migrant workers, foresight tools can support more adaptive and inclusive planning. Based on the findings of this review, we propose a number of recommendations across key sectors.

First, for health systems and the health workforce, we suggest integrating climate-related occupational health into medical, nursing and public health training, with particular attention to HRI recognition and management among migrant worker populations. Additionally, to protect and care for the most at-risk workers, the health sector must establish or strengthen mobile health outreach services capable of reaching workers in remote agricultural and construction settings during extreme weather events.

Second, policymakers and governments must develop and enforce climate-responsive occupational health and safety regulations that explicitly cover migrant workers, including those in informal, seasonal and gig employment. National climate adaptation and disaster preparedness plans need to include migrant workers as a named priority population, with translated materials and community-based communication channels. Moreover, greater investment is needed in health surveillance systems that routinely collect and disaggregate data by occupation and migration status.

Third, employers and the private sector can create immediate change by implementing enforceable heat safety protocols, including rest breaks, hydration, shade and acclimatisation periods, as baseline standards rather than voluntary measures. These types of interventions will be more effective if climate adaptation strategies and emergency response plans are informed by meaningful engagement with workers, particularly migrant workers.

Finally, for researchers and funders, future studies, especially climate-worker-health intervention and preparedness research, must address the dearth of research from low- and middle-income countries, indoor work settings, and non-agricultural sectors. Participatory research approaches that centre the experiences and knowledge of migrant workers themselves will promote accumulation of actionable, accurate knowledge.

This review has several limitations, First, the review only looked at peer review literature, as it was intended to map out the existing literature and identify gaps in research on climate change related preparedness in migrant worker populations. This may result in the inability to capture types of interventions that are more likely to be reported in grey literature than in peer review literature, such as policies implemented by governmental actors or practices adopted by private employers. Second, our strategy included five key concepts (ie, migrant, worker, climate change, health, intervention) and studies were only considered if they included all five concepts. There were instances where relevant papers had to be excluded because they missed one or two criteria. In these cases, we attempted to refer to these studies in the relevant sections of this paper where applicable. Finally, the search was conducted in two databases; the inclusion of additional databases may have identified further relevant studies.

## Conclusions

This scoping review highlights a critical gap in health systems, policy and employer preparedness to protect migrant workers from escalating climate-related health risks. Ultimately, protecting migrant workers’ health amid the growing impacts of climate change will require preparedness strategies grounded in just transition principles to ensure workers have a voice in shaping their rights to social protection, health inclusion and decent work as industries adapt and labour markets evolve in response to climate threats. The most effective multisector preparedness will likely combine migrant inclusive measures, such as universal health coverage and enforceable occupational health protections, with strategic foresight to anticipate climate-related risks. The active participation and leadership of workers is central to building climate-resilient, migrant-inclusive systems that both confront structural inequalities and expand rights, access to health and protections.

## Supplementary material

10.1136/bmjph-2025-003288 online supplemental file 1

## Data Availability

Data sharing not applicable as no datasets generated and/or analysed for this study.
