# Supplementary material for: Perfect storm: a scoping review of interventions and preparedness strategies at the intersections of climate change, migrant worker health and health systems
Source: BMJ Public Health. 2026 Jun 16;4(2):e003288. doi: 10.1136/bmjph-2025-003288 (PMC13289409; doi:10.1136/bmjph-2025-003288)
Supplement: online supplemental file 1 [file bmjph-4-2-s001.pdf]

Supplementary Material

Search strategies used (based on keywords developed from five key concepts in the research question: migrant; worker; climate change; health; preparedness)

Medline

("Transients and Migrants"/ or migrant*.mp. or "Emigrants and Immigrants"/ or immigrant*.mp. or foreign-born.mp. or seasonal.mp. or temporar*.mp. or agricultur*.mp.) and (Occupational Groups/ or worker*.mp. or Employment/ or labour.mp. or labor.mp. or occupation*.mp. or Workplace/ or workplace.mp.) and (Climate Change/ or climate change.mp. or Extreme Heat/ or extreme heat.mp. or heat stress.mp. or Extreme Weather/ or extreme weather.mp. or natural disaster*.mp. or flood*.mp. or wildfire*.mp.) and (Occupational Health/ or health*.mp. or safe*.mp. or injur*.mp. or illness*.mp. or disease*.mp. or death*.mp. or hazard*.mp. or risk*.mp. or exposure*.mp. or hospital*.mp. or surveillance.mp. or emergency medicine.mp.) and (preparedness.mp. or adapt*.mp. or interven*.mp. or polic*.mp. or respons*.mp. or warning system*.mp. or prevent*.mp. or protect*.mp.)

Global Health

(migrants/ or migrant*.mp. or immigrants/ or immigrant*.mp. or foreign-born.mp. or seasonal.mp. or temporar*.mp. or agricultur*.mp.) and (occupations/ or worker*.mp. or employment/ or labour.mp. or labor.mp. or occupation*.mp. or work places/ or workplace.mp.) and (climate change/ or climate change.mp. or heat stress/ or extreme heat.mp. or heat stress.mp. or extreme weather.mp. or natural disaster*.mp. or flood*.mp. or wildfire*.mp.) and (occupational health/ or health*.mp. or safe*.mp. or injur*.mp. or illness*.mp. or disease*.mp. or death*.mp. or hazard*.mp. or risk*.mp. or exposure*.mp. or hospital*.mp. or surveillance.mp. or emergency medicine.mp.) and (preparedness.mp. or adapt*.mp. or interven*.mp. or polic*.mp. or respons*.mp. or warning system*.mp. or prevent*.mp. or protect*.mp.)
